# Combined pressure and volume loading for left ventricular training in patients with congenitally corrected transposition of the great arteries

**DOI:** 10.1016/j.xjon.2024.08.016

**Published:** 2024-09-04

**Authors:** Antonia Schulz, Marcus Kelm, Viktoria H.M. Weixler, Peter Kramer, Mi-Young Cho, Stanislav Ovroutski, Felix Berger, Joachim Photiadis

**Affiliations:** a Department of Congenital and Pediatric Heart Surgery, Deutsches Herzzentrum der Charité, Berlin, Germany; b Charité – Universitätsmedizin Berlin, Corporate Member of Freie Universität Berlin and Humboldt-Universität zu Berlin, Berlin, Germany; c Berlin Institute of Health at Charité – Universitätsmedizin Berlin, Berlin, Germany; d DZHK (German Centre for Cardiovascular Research), Partner Site Berlin, Berlin, Germany; e Department of Congenital Heart Disease – Pediatric Cardiology, Deutsches Herzzentrum der Charité, Berlin, Germany

**Keywords:** ccTGA, enhanced LV training, left ventricular training, l-TGA, PAB, pulmonary artery band

## Abstract

**Objective:**

Patients with congenitally corrected transposition of the great arteries may require left ventricular training before the double switch operation. We evaluated the effects of combined pressure and volume loading.

**Methods:**

We performed a retrospective study of patients with congenitally corrected transposition of the great arteries who underwent left ventricular training between 2012 and 2022.

**Results:**

Fifteen patients underwent left ventricular training at a median age of 1.5 years (interquartile range [IQR], 0.7-5.6). Their median left ventricular mass index was 21 g/m^2^ (IQR, 18.9-36.6), left ventricular end-diastolic volume index was 65.1 mL/m^2^ (IQR, 40.6-84.6), and systolic left ventricular/right ventricular pressure ratio was 0.35 (IQR, 0.31-0.5). In addition to pulmonary artery banding, atrial septectomy was performed in 12 patients (80%). Two patients already had a relevant shunt. One patient required systemic ventricular assist device implantation and heart transplantation. After a median of 1.9 years (IQR, 0.8-4.4), left ventricular mass index had increased to 38.5 g/m^2^ (IQR, 25-49, *P* = .002), left ventricular end-diastolic volume index to 71.4 mL/m^2^ (IQR, 50.1-94.4, *P* = .13), and systolic left ventricular/right ventricular pressure ratio to 0.94 (IQR, 0.84-1.1, *P* = .002). Older patients demonstrated a lower increase in left ventricular pressure. Six patients (6/14, 43%) have met eligibility criteria for the double switch operation (5 performed). Their age at the time of pulmonary artery banding was 1.7 years (IQR, 0.5-3.7), and the time between pulmonary artery banding and double switch operation was 3.1 years (IQR, 1.5-5.2). One patient required double switch operation takedown due to left ventricular failure. Two older patients were considered nonresponders to left ventricular training.

**Conclusions:**

Combined pressure and volume loading resulted in a significant increase in left ventricular mass index and left ventricular pressure. Among older patients, there were nonresponders who remained not suitable for the double switch operation.

## Graphical Abstract

**Figure:**
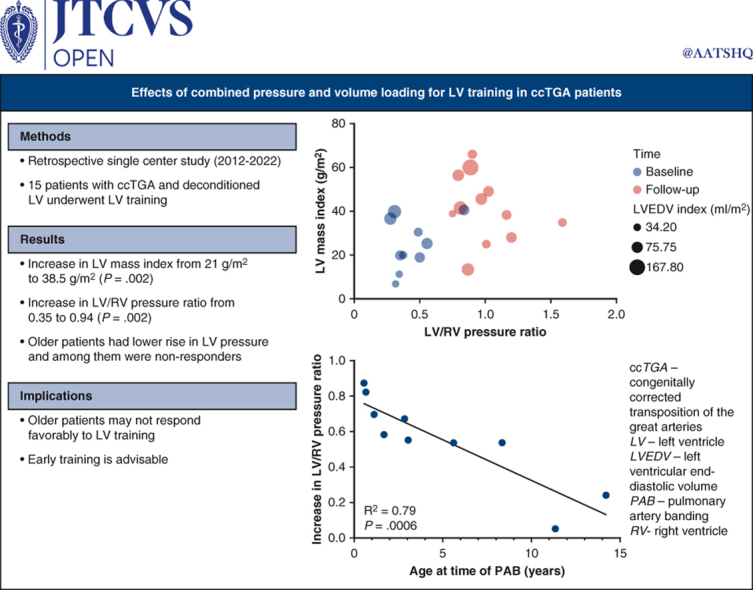

**Figure:**
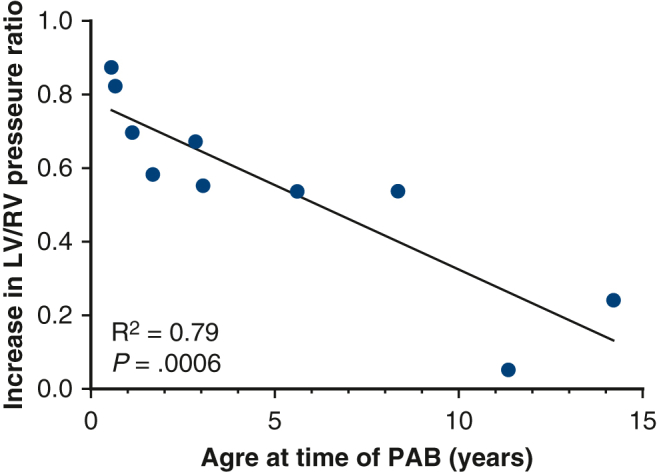
Increase in LV to RV pressure ratio was higher in younger patients.

**Central Message**

LV training with combined pressure and volume loading resulted in a significant increase in LV mass index and LV pressure. Older patients demonstrated a lower increase in LV pressure.

**Perspective**

A subset of patients with ccTGA require LV training before anatomic repair. PAB in combination with an atrial shunt resulted in an increase in LV mass and pressure. Older patients exhibited a lower increase in LV pressure with a higher rate of training failure. Therefore, early start of training is advisable.

In congenitally corrected transposition of the great arteries (ccTGA), the morphologic right ventricle (mRV) supports the systemic circulation. Patients without associated defects may remain asymptomatic until the fourth decade of life when impaired systemic ventricular function can cause congestive heart failure.^1^ In patients with associated lesions, physiologic repairs that leave the mRV in the systemic position have demonstrated unsatisfactory long-term survival and a high incidence of heart failure.^2^, ^3^, ^4^, ^5^ Therefore, the concept of anatomic correction with combined atrial and arterial switch has been pursued as the double switch operation (DSO), which brings the morphologic left ventricle (mLV) into the systemic position and has shown promising midterm results.^6^, ^7^, ^8^, ^9^, ^10^, ^11^, ^12^ However, in the absence of pulmonary stenosis and a large ventricular septal defect (VSD), the mLV is usually unprepared to sustain a systemic workload once pulmonary vascular resistance has decreased during the first weeks of life. Consequently, in patients with ccTGA and intact ventricular septum or with small, restrictive VSDs and without significant left ventricular (LV) outflow tract obstruction, the mLV requires preconditioning before the DSO. Pulmonary artery banding (PAB) increases mLV afterload and has been shown to provide successful retraining of the mLV to undergo anatomic repair at a later stage.^13^, ^14^, ^15^, ^16^ However, establishing a sufficient gradient can be challenging and subsequent PAB adjustments are not uncommon, especially in patients aged more than 1 year.^15^

Zartner and colleagues^17^ have introduced the concept of enhanced ventricular training with combined pressure and volume loading of the mLV by creation of an atrial septal defect (ASD) in addition to PAB. Additional volume loading by an ASD dynamically modulates the gradient across the PAB and may allow for intermittent training and relaxation phases. The ASD also can serve as a pressure-limiting valve to avoid high wall stress with associated myocardial fibrosis. Another potential benefit of volume loading is the promotion of capillary growth, in contrast to myocyte hypertrophy without increasing capillary density, as occurs with isolated pressure loading in older children and may contribute to mLV dysfunction in some of them.^18^, ^19^, ^20^

The purpose of this study was to evaluate the training effects of combined pressure and volume loading in patients with ccTGA and unprepared mLVs.

## Material and Methods

### Study Design

This is a single-center study with retrospective analysis of all patients with ccTGA who underwent PAB for mLV training between 2012 and 2022. Patients who underwent PAB as a palliative procedure without the purpose of mLV training and patients with large nonrestrictive VSDs were excluded. We analyzed the evolution of mLV parameters under combined pressure and volume loading including LV mass and LV end-diastolic volume (LVEDV) evaluated by cardiac magnetic resonance imaging (MRI) and LV to RV systolic pressure ratio measured by cardiac catheterization. The study was approved by the local ethics committee with waived need for informed written consent (EA2/165/19, 15/01/2020). Three international patients were lost to follow-up assessment. In these patients, last available imaging was analyzed.

### Left Ventricle Training

Patients with a diagnosis of ccTGA and unprepared mLV underwent mLV training with the goal of subsequent anatomic repair. A main PAB was placed via sternotomy using a 4-mm wide silastic band adjusted according to Trusler's rule.^21^ The band was then further tightened to increase the LV pressure to one-half to two-thirds of the systemic pressure. Intraoperative echocardiographic assessment was used to ensure preserved LV and mitral valve function with increasing pressure load. In the absence of a significant atrial shunt, an atrial septectomy aiming for a 10-mm atrial communication was performed on cardiopulmonary bypass with the heart fibrillating.

### Left Ventricle Training Assessment

Patients were scheduled for training assessment after 6 to 9 months. Assessment of the mLV training status was done using a combination of echocardiography, MRI, and catheterization. The LV mass index, LVEDV index, mass to volume ratio, LV function, and LV to RV pressure ratio were used to assess readiness for anatomic repair (Table 1). The reassessment of our eligibility criteria for anatomic repairs over time has been previously published.^22^

**Table 1:** Assessment of left ventricular preparedness for anatomic repair

Parameter	Value
LV EF (%)	≥50
LV/RV pressure ratio	≥0.9
LV mass index (g/m^2^)	≥50∗
LVEDV index (mL/m^2^)	40-100
LV end-diastolic pressure	≤12 mm Hg
Mitral valve function	Mild or less insufficiency

*LV*, Left ventricular; *EF*, ejection fraction; *RV*, right ventricular; *LVEDV*, left ventricular end-diastolic volume.

∗ Lower LV mass index was accepted in patients who demonstrated excellent mLV function with suprasystemic mLV pressures.

### Statistical Analysis

Descriptive statistics include mean with SD for normal continuous data, median with interquartile range (IQR) for non-normal continuous data, and frequency with percentage for categorical data. Wilcoxon signed-rank test was used to compare paired variables. Linear regression was used to evaluate the relationship between age at time of LV training commencement and increase in LV mass index and LV to RV pressure ratio. Data analysis was performed with Stata version 18 (StataCorp) and GraphPad Prism version 10.2.2 (GraphPad Software).

## Results

### Baseline Characteristics

Fifteen patients (9/15 female, 60%) with ccTGA underwent LV training at a median age of 1.5 years (IQR, 0.7-5.6) with a median weight of 10 kg (IQR, 7.6-22.2). Before the procedure, 2 patients (13.3%) had an ASD, 2 patients (13.3%) had partial anomalous pulmonary venous drainage, and 3 patients (20%) had a small restrictive VSD. Approximately half of the patients (7/15, 46.7%) were taking heart failure medication before surgery.

The baseline echocardiogram showed tricuspid valve regurgitation was mild or less in 8 patients (8/15, 53.3%), moderate in 3 patients (3/15, 20%), moderate to severe in 1 patient (1/15, 6.7%), and severe in 3 patients (3/15, 20%). Right ventricular (RV) function was preserved in 10 patients (10/15, 66.7%), mildly reduced in 3 patients (3/15, 20%), and moderately reduced in 2 patients (2/15, 13.3%). LV function was preserved in all patients. One patient had mild-moderate mitral valve regurgitation.

Baseline MRI was performed in 11 patients (11/15, 73.3%). The median LVEDV index was 65.1 mL/m^2^ (IQR, 40.6-84.6), left ventricular end-systolic volume index was 17.1 mL/m^2^ (IQR, 11.5-30.3), and LV mass index was 21 g/m^2^ (IQR, 18.9-36.6). The median mass/volume ratio was 0.34 g/mL (IQR, 0.39-0.5), and LV ejection fraction was 69% (IQR, 63.4-74). The median right ventricular end-diastolic volume index was 93.7 mL/m^2^ (67-142.8), right ventricular end-systolic volume index was 37 mL/m^2^ (IQR, 30-66.4), and RV ejection fraction was 57% (IQR, 53.2-60.9). The average pulmonary blood flow:systemic blood flow (Qp:Qs) was 1.1 (IQR, 0.9-1.2). Baseline cardiac catheterization was performed in 12 patients (12/15, 80%). The median systolic LV/RV pressure ratio was 0.35 (IQR, 0.31-0.5).

### Left Ventricle Training Procedure

In addition to placement of a PAB, atrial septectomy was performed in 12 patients (12/15, 80%) to increase LV volume load. Two patients (2/15, 13%) already had a significant shunt due to partial anomalous pulmonary venous drainage. Permanent pacemaker implantation was performed in 3 patients (3/15, 20%) for congenital heart block.

After PAB, the maximum pulmonary artery flow velocity was 3.3 m/s (IQR, 3.1-3.6) with a corresponding maximum gradient of 41 mm Hg (IQR, 40-52). Tricuspid valve regurgitation was mild or less in 9 patients (9/15, 60%), moderate in 5 patients (5/15, 33.3%), and severe in 1 patient (1/15, 6.7%). RV function was preserved in 10 patients (10/15, 66.7%) and moderately reduced in 5 patients (5/15, 33.3%). LV function was preserved in 12 patients (12/15, 80%), and 3 patients (3/15, 20%) had mild dysfunction. No patient had more than mild mitral valve regurgitation.

Median intensive care unit and hospital stay were 1 day (IQR, 1-3) and 6 days (IQR, 5-8), respectively.

### Left Ventricle Training Status

There was no early mortality. One patient decompensated 8.7 months after the procedure and required systemic ventricular assist device (VAD) implantation for RV failure after initial extracorporeal membrane oxygenation support. The patient underwent heart transplantation, but died of fulminant rejection 1.5 years later.

Of the 14 survivors, follow-up MRI and cardiac catheterization were performed in 12 patients (12/14, 85.7%), respectively. Two patients were unable to undergo MRI due to permanent epicardial pacing wires. In these patients, LV volume and muscle mass were measured by echocardiography instead. Median time to last imaging was 1.9 years (IQR, 0.8-4.4).

**Figure 1:**
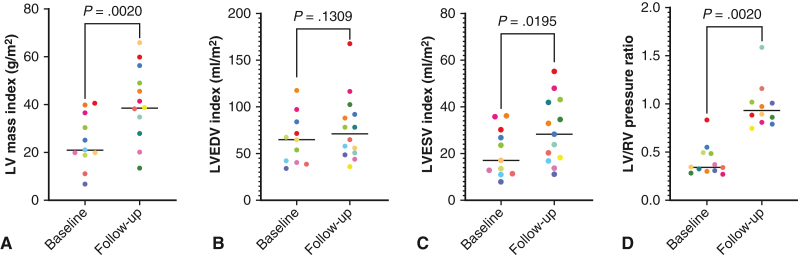
Scatter plot of baseline and follow-up LVEDV indices (A), LVESV indices (B), LV mass indices (C), and LV to RV systolic pressure ratios (D). The *dots* represent individual patient data points. The *horizontal line* represents the median. *LVEDV*, Left ventricular end-diastolic volume; *LVESV*, left ventricular end-systolic volume; *LV*, left ventricle; *RV*, right ventricle.

**Figure 2:**
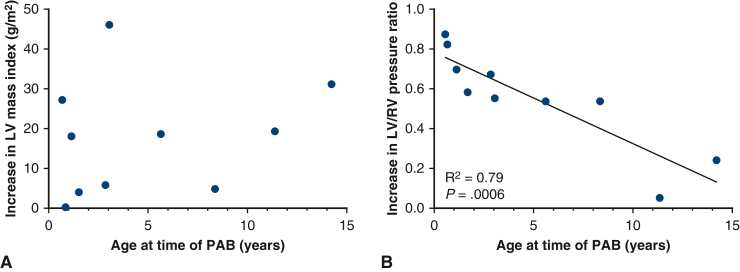
Increase in LV mass index (A) and LV to RV systolic pressure ratio (B) from baseline to last follow-up in patients age 0.4 to 14.2 years. LV/RV pressure ratio increase was higher in younger patients (*P* = .0015, R^2^ = 0.78). The *dots* represent individual patient data points. *LV*, Left ventricle; *RV*, right ventricle; *PAB*, pulmonary artery band.

**Figure 3:**
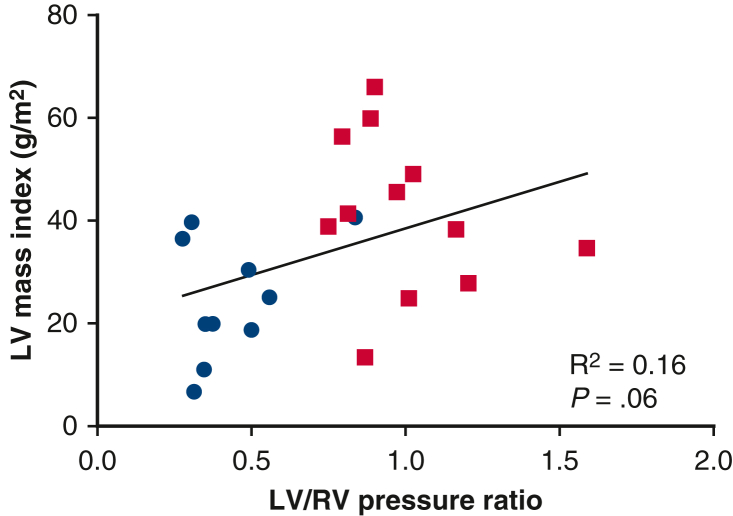
Relationship between LV mass index and LV to RV pressure ratio before LV training (*blue dots*) and at follow-up (*red squares*) (*P* = .06, R^2^ = 0.16). *LV*, Left ventricle; *RV*, right ventricle.

As demonstrated in Figure 1, the median LVEDV index increased to 71.4 mL/m^2^ (IQR, 50.1-94.4, *P* = .13), left ventricular end-systolic volume index to 28.4 mL/m^2^ (IQR, 18.4-41.9, *P* = .02), and LV mass index to 38.5 g/m^2^ (IQR, 25-49, *P* = .002). The median mass/volume ratio was 0.51 g/mL (IQR, 0.36-0.62, *P* = .16), and LV ejection fraction remained at 66% (IQR, 59-69, *P* = .55). The median right ventricular end-diastolic volume index was 81.9 mL/m^2^ (IQR, 66.9-108.2, *P* = .16), right ventricular end-systolic volume index was 42.1 mL/m^2^ (IQR, 22.8-59.2, *P* = .57), and RV ejection fraction remained at 54.5% (IQR, 46.3-59.7, *P* = .3). After atrial septectomy, the average Qp:Qs was 1.8 (IQR, 1.3-2, *P* = .36). Cardiac catheterization revealed a higher systolic LV/RV pressure ratio of 0.94 (IQR, 0.84-1.1, *P* = .002). The median LV end-diastolic pressure was 11 mm Hg (IQR, 9-12.5). On echocardiogram, the maximum PAB gradient had increased to 59 mm Hg (IQR, 52-97, *P* = .003). Although an increase in LV mass index was observed irrespective of the patient's age at the time of the LV training procedure, younger patients demonstrated a higher increase in LV/RV pressure ratio (Figure 2). The relationship between LV mass index and LV to RV pressure ratio is shown in Figure 3.

### Reoperation

After the LV training procedure, 3 patients (3/15, 20%) required unplanned reoperation. One patient (age 8.4 years at time of PAB) required PAB tightening and atrial septectomy 1.2 years after the initial procedure. Atrial septectomy had not been previously performed due to existing shunt from a right PAPVD but without a sinus venous defect. The patient required 2 more procedures: one for external wrapping of the main pulmonary artery secondary to pulmonary artery enlargement below the band and another for size reduction of the ASD. He remains in training 3.9 years after initial procedure.

In 1 patient, LV training strategy was abandoned after 5.4 years and the patient underwent mechanical tricuspid valve replacement, pulmonary artery debanding, closure of a small VSD, and reduction of ASD size. As mentioned above, 1 patient required extracorporeal membrane oxygenation and VAD implantation with subsequent heart transplantation for RV failure 8.7 months after LV training commencement. Another patient is scheduled for PAB tightening 1 year after LV training procedure.

### Double Switch Operation

So far, 6 patients (6/14, 42.9%) have met eligibility criteria for DSO. Their median age at the time of PAB had been 1.8 years (IQR, 0.6-3.1). Five procedures (5/14, 35.7%) have been performed, and 1 is scheduled.

The median age at the time of DSO was 6 years (IQR, 5.8-7.2) with a median time between PAB and DSO of 3.1 years (IQR, 1.5-5.2). Before DSO, median LV mass index was 45.6 g/m^2^ (IQR, 34.8-49), median LVEDV index was 78.3 mL/m^2^ (IQR, 56-78.4), median LV mass to volume ratio was 0.62 g/mL (IQR, 0.52-0.69), median LV ejection fraction was 50% (IQR, 45.5-57.5), and systolic LV to RV pressure ratio was 1.02 (IQR, 0.97-1.2).

Three patients presented with coronary anomalies: 1 with single coronary ostium, 1 with left anterior descending artery from the right coronary artery, and 1 with all coronary arteries arising from a single sinus. One patient had dextrocardia. DSO was performed with a modified Senning atrial switch in all but 1 patient who underwent a hemi-Mustard with Glenn anastomosis. The median cardiopulmonary bypass time was 391 minutes (IQR, 274-505) with a median aortic crossclamp time of 211 minutes (IQR, 162-245). There was no mortality.

One patient had postoperative LV failure and underwent cardiopulmonary resuscitation with extracorporeal membrane oxygenation implantation and DSO takedown after 3 days of which he recovered uneventfully. This patient had a myocardial biopsy performed 2 years previously, which revealed a lymphocytic inflammatory reaction. One patient required temporary mechanical circulatory support that was successfully weaned with uneventful further recovery. One patient required early reoperation with left internal thoracic artery to left anterior descending artery bypass grafting due to coronary stenosis. Median intensive care unit stay was 11 days (IQR, 10-13), and median hospital stay was 21 days (IQR, 16-23). After a median follow-up time of 2.4 years after DSO (IQR, 1.1-2.9), median LV ejection fraction was 48% (IQR, 37.5-56.5) with 3 patients in New York Heart Association (NYHA) class I and 1 patient in NYHA class II. One patient had moderate neoaortic valve regurgitation.

### Failed Left Ventricle Training

There were 2 patients who were considered nonresponders to LV training. They were aged 11 years and 14 years at the time of PAB. One patient eventually underwent debanding and ASD reduction together with mechanical tricuspid valve replacement and closure of a small VSD. The other patient remained with a palliative PAB. Both patients are in NYHA class II with preserved cardiac function 5.5 and 4.6 years after the LV training procedure. Table 2 summarizes individual LV training status and outcomes of all patients.

**Table 2:** Individual training parameters and outcome for all 15 patients who underwent left ventricular training

Patient	Age at time of PAB (y)	Baseline LV mass index (g/m^2^)	Baseline LVEDV index (mL/m^2^)	Baseline systolic LV/RV pressure ratio	LV training duration (y)	Last LV mass index (g/m^2^)	Last LVEDV index (mL/m^2^)	Last systolic LV/RV pressure ratio	Outcome
1	0.4	-	-	-	1.3	38.8∗	36∗	0.75	In training
2	0.5	-	-	-	0.3	34.8	50.5	1.59	Successful DSO
3	0.6	-	-	0.33	4.6	27.9	78.3	1.2	Successful DSO
4	0.7	11.1	38.7	0.34	4.4	38.3	64.6	1.16	DSO scheduled
5	0.8	19.9	40.6	0.38	0.5	20.1	44.2	-	Lost to FU
6	1.1	6.8	34.2	-	0.7	24.9	48.9	1.01	Lost to FU
7	1.5	18.9	67.6	0.5	0.7	-	-	-	ECMO, VAD, heart transplant, death
8	1.5	21	42	-	0.8	25	58	-	In training
9	1.7	-	-	0.29	1.2	13.4	102.3	0.87	Lost to FU
10	2.9	39.8	117.6	0.31	2.3	45.6	88	0.97	Successful DSO with Glenn
11	3.1	19.9	65.1	0.35	5.9	66∗	56∗	0.9	Failed DSO with DSO takedown
12	5.6	30.4	54.2	0.49	1.4	49	78.4	1.02	Successful DSO
13	8.4	36.6	97.1	0.28	3.1	41.4	116.7	0.81	In training (after PAB and ASD adjustment)
14	11.4	40.6	71.8	0.84	5.3	59.9	167.8	0.88	Failed training
15	14.2	25.2	84.6	0.56	3	56.4	91.8	0.8	Failed training

*PAB*, Pulmonary artery banding; *LV*, left ventricle; *LVEDV*, left ventricular end-diastolic volume; *RV*, right ventricle; *DSO*, double switch operation; *FU*, follow-up; *ECMO*, extracorporeal membrane oxygenation; *VAD*, ventricular assist device.

∗ Measured by echocardiography.

## Discussion

Patients with ccTGA and unprepared morphologic LVs require training before DSO. Placement of a PAB increases mLV afterload and stimulates the ventricle to increase muscle mass. Over time, the mLV should be able to build up systemic pressure. Although there is no consensus on exact thresholds to predict successful anatomic correction after LV training, parameters such as LV mass index, LVEDV index, LV mass/volume ratio, LV/RV pressure ratio, and LV function are taken into account to make individual treatment decisions.^12^^,^^14^ Successful retraining of the mLV by PAB alone has been demonstrated with 40% to 75% of patients subsequently able to undergo anatomic correction, but the need for PAB adjustment during LV training is not uncommon.^13^, ^14^, ^15^, ^16^

Additional volume loading of the mLV by creation of an atrial shunt as described by Zartner and colleagues^17^ could provide several benefits over LV training with PAB alone: The dynamic modulation of the PAB gradient during physical activity creates an intermittently elevated LV strain rather than continuous ventricular wall stress, which potentially allows for periods of myocardial regeneration.^17^^,^^23^ The atrial connection also can serve as a pop-off valve in the event of a sudden, very high, and potentially damaging LV load.^17^ However, cardiopulmonary bypass is required for ASD creation of a certain size. Zartner and colleagues^17^ demonstrated success of this concept in a series of 6 patients of whom 4 had failed conventional training by PAB alone. All patients showed an increase in LV mass and volume and achieved systemic LV pressures within 1 year of training. They all underwent successful DSO after a median training time of 1.2 years.

**Figure 4:**
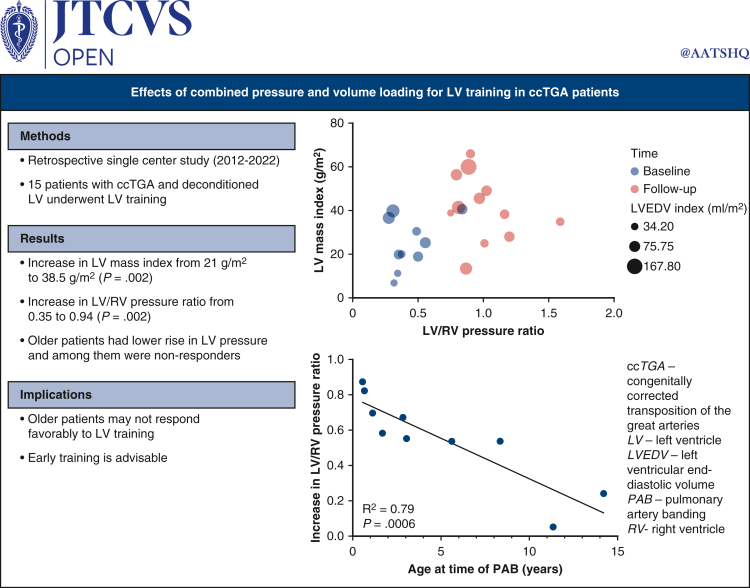
Effects of combined pressure and volume loading for LV training in patients with ccTGA.

In our cohort, 15 patients underwent LV training with additional creation of an ASD in 12 patients, and 2 patients had a shunt due to right partial anomalous pulmonary venous drainage. One patient required systemic VAD support 8 months after PAB due to cardiac decompensation in the context of a viral gastrointestinal infection. The patient died of fulminant rejection several years after successful heart transplantation. In the remaining 14 patients, we observed effects of mLV training with an increase in LV mass index to 38.5 g/m^2^, LVEDV index to 71.4 mL/m^2^, and a mass to volume ratio of 0.51 g/mL after a median training time of 1.9 years. In patients who underwent invasive assessment, the average systolic LV to RV pressure ratio was almost systemic with 0.94. In contrast to the report of Mainwaring and colleagues^15^ who have described a consistent relationship between LV pressure and mass, this was not the case in our series. Although we observed a variable increase in muscle mass across all ages, the increase in LV/RV pressure ratio was lower in older patients (Figure 4). This finding would be another indicator that older patients seem more likely to react with pathological muscle hypertrophy in response to PAB that does not allow them to produce an appropriate increase in pressure despite increase in muscle mass.

Toba and colleagues^20^ have recently demonstrated in their histopathological analysis of patients with TGA, intact ventricular septum, and PAB, an approximately 50% larger myocyte diameter with 20% lower capillary density compared with normal cardiac anatomy, which might contribute to LV dysfunction after anatomic repair. In contrast, patients with TGA/VSD had normal or even increased capillary density with normal myocyte size, which underlines the potential beneficial effect of additional volume loading in LV training.^20^ However, it remains unclear up to what age these effects could be stimulated by an mLV training with combined pressure and volume loading.

Pathological hypertrophy was also discussed by the Stanford group as the underlying cause in patients who did not follow a predictable relationship of LV mass and pressure. They found 3 outliers in their series all of older age.^15^ The 2 oldest patients in our series (age 11 and 14 years) were nonresponders to combined pressure and volume loading. The third oldest patient (age 8 years at time of PAB) remains in training after 3 years, but required multiple adjustments including PAB tightening and ASD creation due to insufficient training response, wrapping of the pulmonary artery to increase PAB efficiency, and reduction of ASD size to 6 mm due to high LVEDV. In this patient, the right amount of volume load was more difficult to judge because the patient also had partial anomalous pulmonary venous drainage. Therefore, despite good overall improvement in LV parameters, not all patients seem to respond favorably to enhanced LV training, particularly older patients. However, despite a higher rate of training failure in older patients, it remains difficult to recommend an absolute age cutoff because there are reports of patients who underwent subsequent DSO at 21 years of age.^14^ Yet, there is consensus that patients should undergo mLV training as early as possible if an anatomic repair shall be pursued.^13^^,^^16^^,^^24^

So far, 6 patients have met eligibility criteria for DSO in our cohort. Unfortunately, 3 international patients are lost to follow-up assessment, and we ultimately cannot assess their readiness for DSO. Five patients have undergone DSO with 1 patient requiring early take down due to LV failure. In this patient, DSO was performed approximately 6 years after the beginning of LV training at age 3 years once the patient had reached a sufficient LV mass index of 66 g/m^2^ and 90% systemic LV pressure. Except for the patients aged 9 years, there were no risk factors for DSO failure. However, the patient did have an abnormal myocardial biopsy 2 years previously demonstrating lymphocytic inflammation. The oldest patient in our series who underwent successful DSO was 5.6 years old at the time of PAB and atrial septectomy. This compares to the series of Zartner and colleagues,^17^ who have been able to successfully train all their patients (oldest 5.6 years, remaining patients aged <3 years) with combined pressure and volume loading.

Despite the theoretical advantages of enhanced mLV training, other groups have demonstrated good results with PAB alone, which avoids the use of cardiopulmonary bypass, although repeat PAB might be necessary especially in older patients. In the recent update of the Stanford mLV training series, they have shown a high success rate with so far 45 of 61 patients (73%) who underwent successful DSO with a bidirectional Glenn anastomosis in 39 of 45 patients (87%).^25^ Approximately 60% of their patients required more than 1 PAB.^25^ After DSO, more than 90% of their patients had preserved LV function.^25^ However, they also report 5 patients with unsuccessful training despite several PAB.^25^ Those patients were older compared with training responders and ranged from age 4 to 17 years.^25^

Therefore, the success rate of mLV training with PAB alone or in combination with an ASD is influenced by patient's age. The addition of an atrial shunt did not completely eliminate the need for repeat training procedures in our series. However, the cohort demonstrated favorable training parameters. Whether the theoretical benefits of an atrial shunt with controlled volume loading will translate into improved clinical outcomes remains to be seen.

### Limitations

This study is limited by its retrospective design, and generalization of the results is not possible because we analyzed only a small cohort of patients. Because mLV training for patients with ccTGA remains a rare procedure, the results still add valuable information to the existing literature where data on enhanced mLV training are scarce. MRI is the gold standard for assessing volume and muscle mass. The use of echocardiographic measurements in patients who were unable to undergo MRI assessment may lead to deviations. Furthermore, comparison of LV training parameters to reference values from the normal population was not possible because there are no suitable percentiles for cardiac MRI data in young children.

## Conclusions

Combined pressure and volume loading resulted in a significant increase in LV mass index and LV/RV pressure ratio. Older patients demonstrated a lower increase in LV pressure, and among them were nonresponders who remained not suitable for anatomic repair. Therefore, early start of training is advisable.

### Webcast

You can watch a Webcast of this AATS meeting presentation by going to: https://www.aats.org/resources/combined-pressure-and-volume-l-7319.

**Figure:**
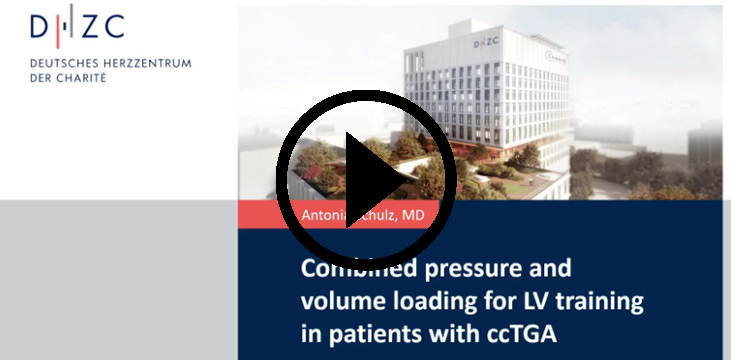

## Conflict of Interest Statement

The authors reported no conflicts of interest.

The *Journal* policy requires editors and reviewers to disclose conflicts of interest and to decline handling or reviewing manuscripts for which they may have a conflict of interest. The editors and reviewers of this article have no conflicts of interest.
